# Overexpression of miRNA-497 inhibits tumor angiogenesis by targeting VEGFR2

**DOI:** 10.1038/srep13827

**Published:** 2015-09-08

**Authors:** Yingfeng Tu, Li Liu, Dongliang Zhao, Youbin Liu, Xiaowei Ma, Yuhua Fan, Lin Wan, Tao Huang, Zhen Cheng, Baozhong Shen

**Affiliations:** 1 Key Laboratory of Molecular Imaging, College of Heilongjiang Province, Harbin, Heilongjiang, China; 2 Department of Cardiology, the Fourth Hospital of Harbin Medical University, Harbin, Heilongjiang, China; 3 Department of Anesthesiology, the Third Hospital of Harbin Medical University, Harbin, Heilongjiang, China; 4 Department of Radiology, the Fourth Hospital of Harbin Medical University, Harbin, Heilongjiang, China; 5 Department of Cardiology, the Second Hospital of Harbin Medical University, Harbin, Heilongjiang, China; 6 Molecular Imaging Program at Stanford, Department of Radiology and Bio-X Program, Stanford University, Stanford, California, USA; 7 College of Pharmacy, Harbin Medical University, Daqing, Heilongjiang, China

## Abstract

Recent studies reported miR-497 exhibited inhibitory effects in various cancers. However, whether miR-497 is involved in inhibiting angiogenesis, which is critical for tumor growth and metastasis, is still unknown. The purpose of this study was to investigate the potential role of miR-497 in tumor angiogenesis. In this work, cell proliferation and apoptosis analyses were conducted to explore the potential function of miR-497 in HUVECs by using MTT and TUNEL assays. Western blotting (WB) was employed to validate the downstream targets of miR-497. Furthermore, in order to disclose the role of miR-497 on angiogenesis, VEGFR2-luc transgenic mice were treated with miR-497 mimic and applied to monitor tumor angiogenesis and growth by *in vivo* bioluminescent imaging (BLI). The results demonstrated that overexpression of miR-497 showed inhibitory effects on VEGFR2 activation and downstream Raf/MEK/ERK signal pathways *in vitro* and *in vivo*. Moreover, overexpression of miR-497 effectively induced HUVECs apoptosis by targeting VEGFR2 and downstream PI3K/AKT signaling pathway. Furthermore, miR-497 exhibited anti-angiogenesis and anti-tumor effects in the VEGFR2-luc breast tumor model proven by BLI, WB and immunohistochemistry analysis. In summary, miR-497 inhibits tumor angiogenesis and growth via targeting VEGFR2, indicating miR-497 can be explored as a potential drug candidate for cancer therapy.

Angiogenesis is critical for tumor growth and progression, because it forms a network of blood vessels which penetrate into cancer growth to supply nutrients and oxygen and to remove metabolic waste from the tumor. Therefore, tumor growth and metastasis are highly dependent upon the angiogenic process^1^, implying that anti-angiogenic drugs may be important in the cancer therapy^2^. Indeed, anti-angiogenic agents have been proven to inhibit the growth of a wide variety of solid tumors in animal models by reducing tumor blood supply^3^.

Among a series of angiogenic inducers, vascular endothelial growth factor (VEGF) is probably the most critical one^4^, and a large body of evidence suggests that VEGFR-2 is the most important of the VEGF receptors with regard to tumor angiogenesis^5^,^6^. Endothelial cells play a pivotal role in each step of pathological angiogenesis, including cell migration, proliferation, invasion, adhesion, and tube formation^7^. VEGF, the most essential factors regulating angiogenesis, is mainly associated with VEGFR2-mediated signal transduction in endothelial cells^8^. In general, binding of VEGF to the VEGFRs activates several intracellular signaling molecules, such as phosphatidylinositol 3-kinase/Akt and mitogen-activated protein kinases (MAPKs) in endothelial cells^9^, which promotes angiogenic growth and maturation. Among the endothelial cell signaling pathways that regulates endothelial cell migration, proliferation, growth and survival, phosphoinositide 3-kinase (PI3K)/Akt and Raf/MEK/ERK pathways are the major pathways. The activation of these two pathways in endothelial cells is necessary for tumor angiogenesis^10^,^11^. Evidence from recent studies has demonstrated that Akt and ERK activated and promoted endothelial cell proliferation by stimulating VEGF. For example, Jin *et al*. reported that VEGF-induced choroid-retinal endothelial cells proliferation and tube formation required PI3K-Akt and Raf/MEK/ERK signaling pathways, and both of the two pathways were needed in regulating VEGF expression^12^. Silvestre *et al*. demonstrated that VEGF promotes angiogenesis through the phosphorylation of Akt and ERK in endothelial cells of mouse ischemic limb^13^, indicating that Akt and ERK genes maybe play key roles in angiogenesis pathways. In addition, the PI3K/Akt pathway could also play significant role in tumors, which is one of the most commonly activated signaling pathways and is well known to be a major cell survival pathway in many cancers^14^,^15^.

MicroRNAs (miRNAs), a subset of small single-strand, non-coding RNAs, have been identified as important regulators that modulate target gene expression post-transcriptionally in a wide variety of oncogenic processes^16^. Aberrant miRNA expression has been shown to be crucial in many human malignancies^17^. Recent studies have indicated that expression of some miRNAs is closely correlated with cancer development and some miRNAs are known to have diverse functions in regulating biological processes, including development, cell proliferation, differentiation, apoptosis, and cancer initiation or progression^18^,^19^,^20^. In the past decade, a number of miRNAs have been found to be aberrantly expressed in angiogenesis-associated endothelial cells, and the roles of miRNAs in tumor angiogenesis have also been extensively disclosed^21^,^22^,^23^. Initially, miR-221 and miR-222 are known to modulate the angiogenic properties of human umbilical vein endothelial cells, forced expression of miR-221/222 represses c-kit expression and also inhibits endothelial cell-facilitated tube formation^24^. Next, Yang *et al*. demonstrated the important role of miR-93 in tumorigenesis and angiogenesis by targeting integrin-beta8^25^. Moreover, Lee *et al*. revealed that overexpression of miR-378 promoted tumorigenesis and angiogenesis by acting on SuFu and Fus-1 expression^26^. Recently, some studies showed that the aberrant expression of miR-497 was associated with proliferation and apoptosis in various kinds of human cancers, and miRNAs profiling studies demonstrated that miR-497 had the potential functions in neuroblastoma^27^, gastric cancer^28^, colorectal cancer^29^, cervical cancer^30^, and breast cancer^17^,^31^.

However, till now, the regulating role of miR-497 in tumor angiogenesis is far from fully elucidated. By bioinformatics, we showed that the 3′UTR of VEGFR2 contains a potential binding element for miR-497 with a 7-nt matching to the miR-497 seed region. To this end, we hypothesized that miRNA-497 may have a role in regulating VEGFR2 expression at the posttranscriptional level and that the dysregulation of miRNA-497 might contribute to the onset of tumor angiogenesis. In this study, we explored that miR-497 regulated tumor angiogenesis by targeting VEGFR2 and its downstream signaling pathway *in vitro* and *in vivo*. Accordingly, this study indicated that manipulating the expression of miR-497 represents a promising therapeutic strategy for tumor angiogenesis.

## Results

### VEGFR2 is the target gene of miR-497 in HUVECs

**Figure 1:**
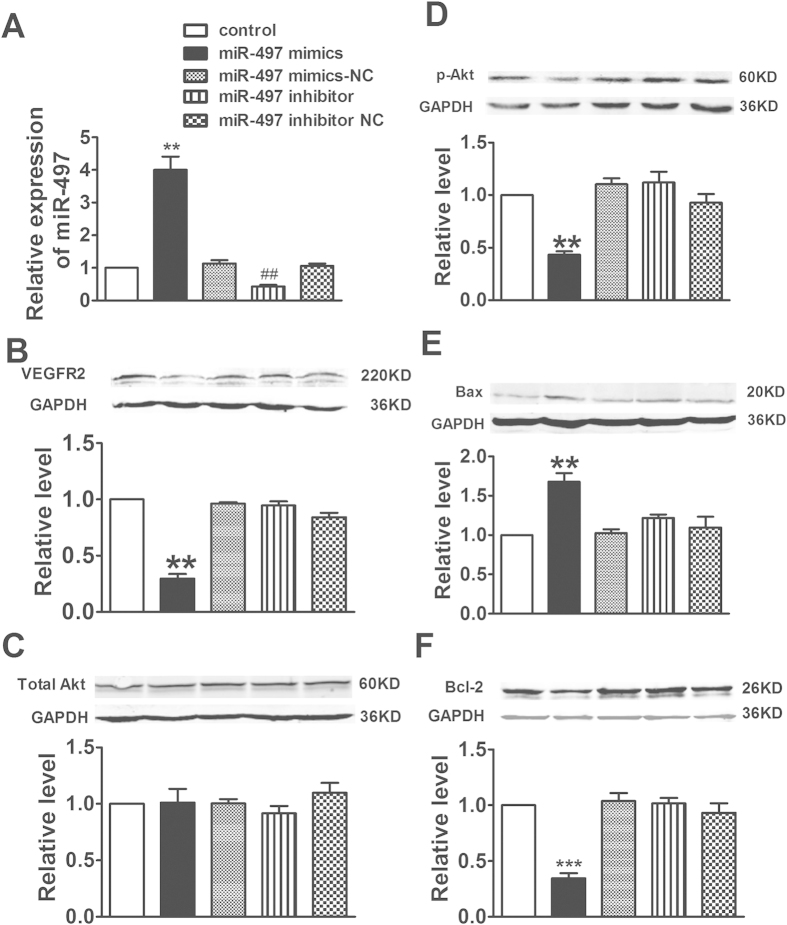
Up-regulation of miR-497 mitigated VEGFR2 level and increased the levels of apoptosis related proteins in human umbilical vein endothelial cells (HUVECs). (**A**) Using qRT-PCR analysis, the expression of miR-497 was increased in miR-497 mimic treatment and decreased in miR-497 inhibitor treatment in HUVECs. Data were expressed as mean ± SEM, n = 3; ^**^P < 0.01 *vs*. control group, ^##^*P *< 0.01 *vs*. control group. (**B–F**) Protein levels of VEGFR2, Total Akt, p-Akt, Bax, and Bcl-2 were detected by western blotting assay. Data were expressed as mean ± SEM, n = 3; ^**^P < 0.01 or ^***^P < 0.001 *vs*. control group.

One of the predicted targets of miR-497 is VEGFR2 by using the specific program TargetScan (http://www.targetscan.org) (see Supplementary material online, Fig. S1A). In order to further demonstrate that miR-497 may inhibit VEGFR2 expression through direct interaction with predicted binding sites located in the 3′-UTR region of VEGFR2 in HUVECs, we cloned a reporter vector in which luciferase cDNA was followed by a fragment of the 3′-UTR from VEGFR2 mRNA containing the putative miR-497 binding sequences. Furthermore, we synthesized another luciferase reporter fused to the VEGFR2 3′-UTRs, but with a mutant miR-497 binding sequence. We then transfected this luciferase reporter vector with either wild-type or mutant miR-497 binding sequences into HUVECs. We also co-transfected these cells with miR-497 mimic or mimic control and measured luciferase activity. As shown in Fig. 1B, the miR-497 mimic administered at a concentration of 50 nM decreases luciferase activity of the reporter vector containing miR-497 binding sequences (see Supplementary material online, Fig. S1B). However, the miR-497 mimic has no significant effect on the reporter vector with mutated miR-497 binding sequences (see Supplementary material online, Fig. S1C). These data suggest that miR-497 may inhibit translation of VEGFR2 by directly acting on response elements specific for miR-497 in the VEGFR2 3′-UTR region in HUVECs.

### Overexpression of miR-497 induced apoptosis of HUVECs and 4T1 cells via targeting VEGFR2

HUVECs and 4T1 breast cancer cells were transfected with 100 nmol/L of miR-497 mimic, miR-497 mimic-NC, miR-497 inhibitor, or miR-497 inhibitor-NC for 24 h. miR-497 expression was evaluated by real-time quantitative PCR after transfection. The results showed that miR-479 mimic increased while miR-497 inhibitor decreased the expression of miR-497 in HUVECs compared with the control group (*P *< 0.05). No statistical significance of miR-497 expression was found among the control, miR-497 mimic-NC and miR-497 inhibitor-NC groups (*P *> 0.05) (Fig. 1A).

We then tested the influence of miR-497 on the expression of VEGFR2 and its downstream proteins Akt, Bcl-2 and Bax in HUVECs, which were related to apoptosis. The western blotting results showed that treatment of HUVECs with miR-497 mimic significantly decreased VEGFR2 and its downstream proteins p-AKT, Bcl-2 expression, while increased Bax expression, demonstrating that miR-497 mimic significantly inhibited the angiogenesis responses induced by HUVECs (Fig. 1B–F). However, the total Akt expression was not influenced in all groups (Fig. 1C). These data demonstrate that overexpression of miRNA-497 inhibit the survival of HUVECs via targeting VEGFR2 and its downstream signal pathway. However, the decreased degree of miR-497 in HUVECs after miR-497 inhibitor treatment was not obvious, only down to 50% relative to control group. So, no obvious up-modulation of targets and related pathways changes in subsequent were observed.

Next, the effect of miR-497 on apoptosis in HUVECs using TUNEL analysis was investigated. Data from TUNEL assay showed that the percentage of apoptosis of each group was as follows: control (6.3 ± 0.5%), miR-497 mimic (35.6 ± 1.2%), mimic-NC (7.2 ± 0.8%), inhibitor (5.2 ± 0.3%), and inhibitor-NC (6.6 ± 0.7%) (see Supplementary material online, Fig. S2). From the above data, we concluded that up-regulation of miR-497 markedly increased HUVECs apoptosis compared to control group (*P *< 0.05) (see Supplementary material online, Fig. S2).

In addition, we also found that miR-479 mimic increased the expression of miR-497 in 4T1 breast cancer cells compared with the control group (*P *< 0.05) (see Supplementary material online, Fig. S4A). And overexpression of miRNA-497 inhibited the survival of 4T1 breast cancer cells via targeting VEGFR2 and its downstream signal pathway proteins Bcl-2 and Bax expression (see Supplementary material online, Fig. S4B–D), which markedly increased 4T1 breast cancer cells apoptosis (see Supplementary material online, Fig. S4E–F). Taken together, these results suggested that miR-497 could act as an apoptosis inducer in HUVECs and 4T1 breast cancer cells *in vitro*.

### miR-497 modulates HUVECs proliferation via targeting VEGFR2 and its downstream Raf/MEK/ERK signal pathway

**Figure 2:**
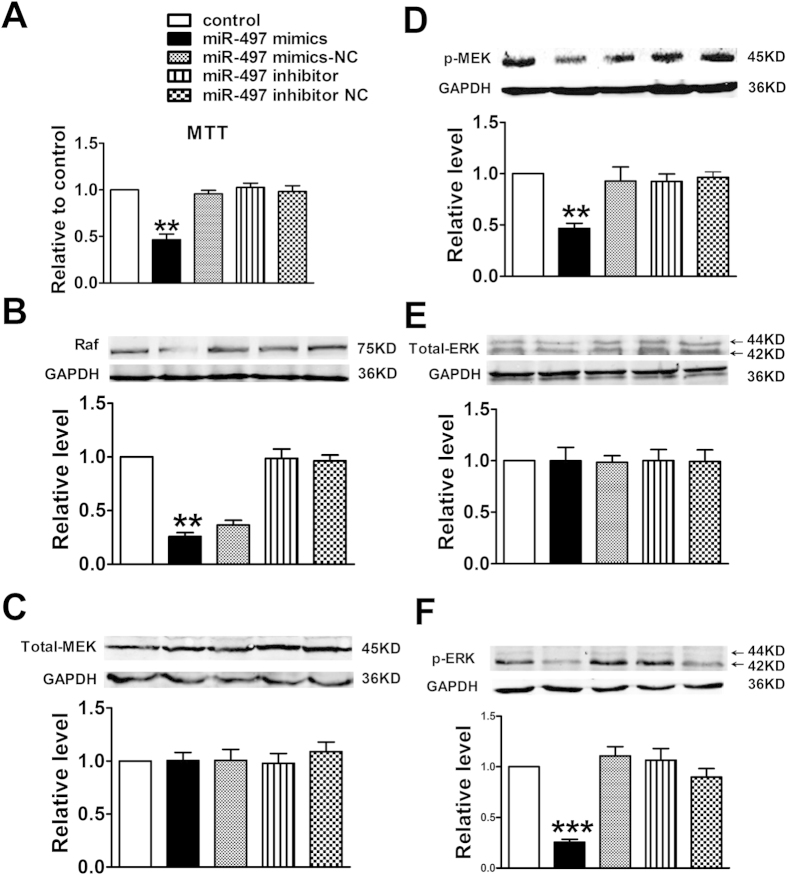
Overexpression of miR-497 influenced the proliferation of cultured HUVECs. (**A**) Cell proliferation was evaluated by the MTT assay; (**B**–**F**) Protein levels of Raf, Total-MEK, p-MEK, Total-ERK and p-ERK were determined by western blotting assay. Data were expressed as mean ± SEM, n = 3; ^**^P < 0.01 or ^***^P < 0.001 *vs*. control group.

Angiogenesis is a complex process orchestrated by both growth factors and cell adhesion, and it is initiated by focal degradation of the vascular basement membrane with subsequent migration and proliferation of endothelial cells. The Raf/MEK/ERK pathway is required for endothelial cell function during angiogenesis. To explore the potential impact of miR-497 on the proliferation of HUVECs, cellular viability and proliferation were measured following the protocol of MTT and western blotting assay. The results showed that HUVECs transfected with miR-497 mimic, but not miR-497 inhibitor, proliferated at a lower rate as compared with control group (*P *< 0.05) (Fig. 2A), indicating that up-regulation of miR-497 could suppress the growth of HUVECs. However, miR-497 mimic-NC could not play the same role (*P *> 0.05) (Fig. 2A).

To further confirm the role of miR-497 in the anti-proliferative action in HUVECs, we performed gain- and loss-of-function studies on the expression of miR-497 using *in vitro* cells transfection. The results showed that transfection of the miR-497 mimic led to a significant decrease in the expression of Raf, p-MEK, and p-ERK proteins in HUVECs (*P *< 0.05) (Fig. 2B,D,F), accompanied by parallel down-regulation of VEGFR2 expression. In concordance with our expected, miR-497 mimic had no effect on total MEK and ERK proteins expression (*P *> 0.05) (Fig. 2C,E). Taken together, these results demonstrated that up-regulation of miR-497 was capable of inhibiting the proliferation of HUVECs via targeting VEGFR2/Raf/MEK/ERK signal pathway.

### Overexpression of miR-497 inhibits angiogenesis in 4T1 tumor bearing mice using BLI *in vivo*

**Figure 3:**
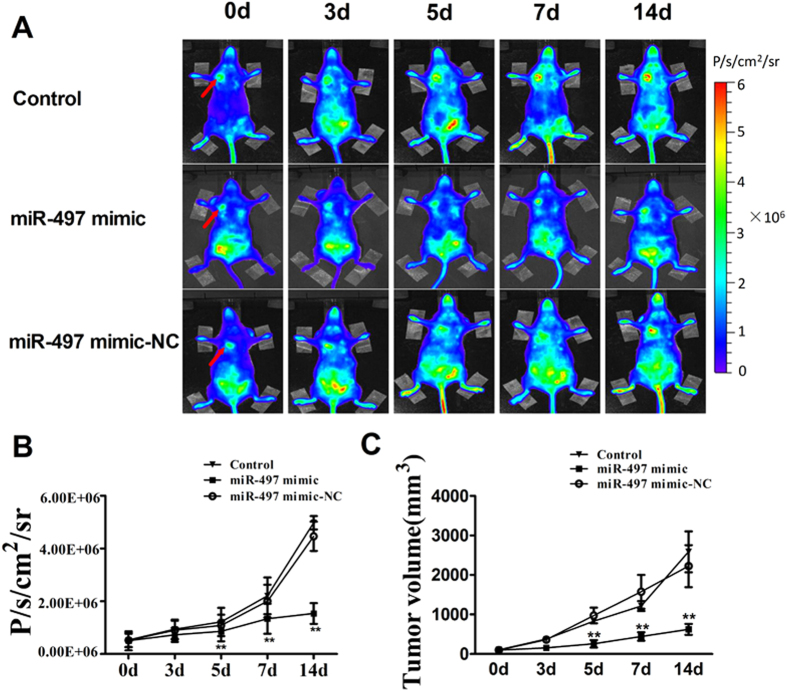
Bioluminescent imaging of tumor angiogenesis and alterations of tumor volume. (**A**) Bioluminescent imaging of tumor-bearing mice was obtained at 0 d, 3 d, 5 d, 7 d and 14 d in the same imaging conditions. (**B**) The dynamic measurements of bioluminescent intensity in tumors treated with saline, miR-497 mimic and miR-497 mimic-NC. Regions of interest (ROI) from displayed images revealed on the tumor sites and quantified as maximum photons per second per centimeter squared per steradian (P/s/cm^2^/sr). Data were expressed as mean ± SEM, n = 5; ^**^P < 0.01 *vs*. control group. (**C**) Tumor volume curves of mice treated with saline, miR-497 mimic and miR-497 mimic-NC. Data were expressed as mean ± SEM, n = 5; ^**^P < 0.01 *vs*. control group.

For real-time visualization of the anti-angiogenesis effects of miR-497 via targeting VEGFR2, we used VEGFR2-luc transgenic mice to monitor tumor growth and angiogenesis *in vivo* by using BLI. After tumor cells injection for seven days, the VEGFR2-luc transgenic mice with tumor were randomized into three groups with the similar mean tumor volume: control group, miR-497 mimic group, miR-497 mimic-NC group. We defined the miR-497 mimic treatment day as 0 d, and as shown in Fig. 3A, there was no significant difference of tumor volume and bioluminescence signal intensity among three groups at 0 d (*P *> 0.05). With the increasing of tumor size, the bioluminescence signal was gradually enhanced in different treatment groups. Compared with the control, the bioluminescence signal intensity of miR-497 mimic treatment group was markedly lower on 5 d (*P *< 0.05) (Fig. 3A,B). Furthermore, the reduction of bioluminescence signal intensity was observed in miR-497 mimic group (~76%) compared to control on 14 d (*P *< 0.05). However, there was no statistical difference observed between control and miR-497 mimic-NC from 0 d to 14 d (*P *> 0.05) (Fig. 3A,B).

The similar results appeared in the changes of tumor volumes. There was no obvious difference in tumor size of three animal groups at 0 d (*P *> 0.05) (Fig. 3C). However, tumor sizes of miR-497 mimic treatment group were found to be smaller than those in control at 5 d (*P *< 0.05) (Fig. 3C), which was much more apparent on 14 d (Fig. 3C). These results indicated that VEGFR2-luc mouse was a useful tool which could monitor the therapeutic effect of anti-angiogenesis *in vivo*. And overexpression of miR-497 played an important role in suppressing tumor angiogenesis and inhibiting tumor growth with down-regulation of VEGFR2.

### Up-regulation of miR-497 inhibits tumor growth by inducing cell apoptosis

**Figure 4:**
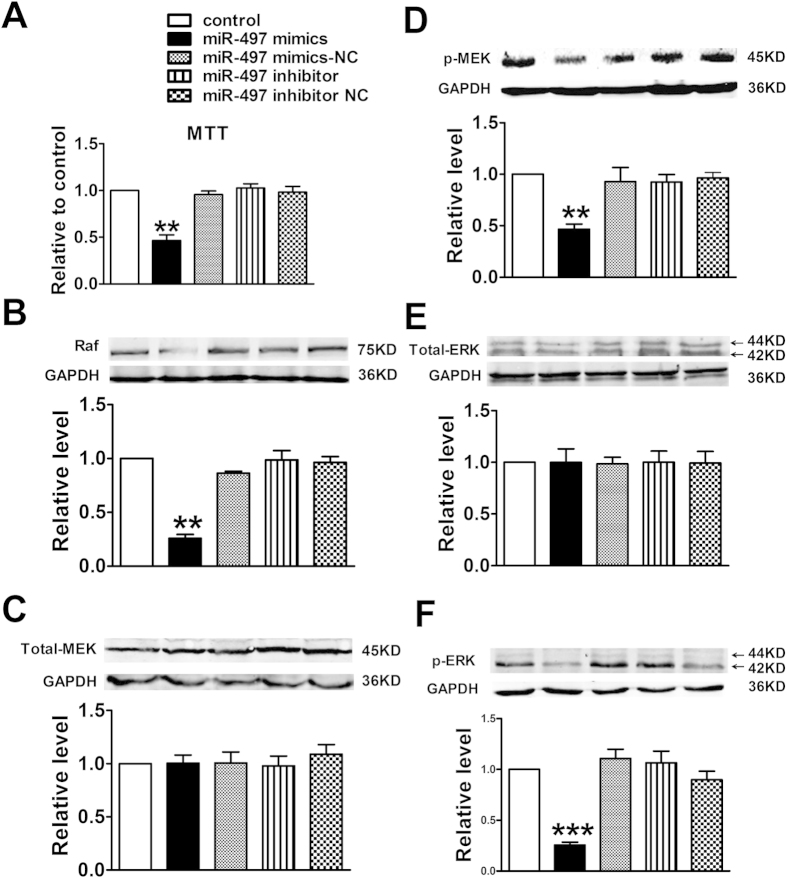
Effects of miR-497 overexpression on apoptosis in mouse breast tunmor model. (**A**) Quantitative analysis of miR-497 expression in tumor tissues by qRT-PCR analysis; (**B**–**D**) Proteins expression of VEGFR2, Bcl-2, and Bax. Data were expressed as mean ± EM, n = 3; ^**^P < 0.01 *vs*. control group. (**E**) Cell apoptosis was determined by TUNEL assay and shown as percentage. (**F**) Representative apoptotic images of each group were taken at a magnification of ×200. Data were expressed as mean ± SEM, n = 10; ^**^P < 0.01 *vs*. control group.

The mice were sacrificed and tumor tissues were removed after *in vivo* bioluminescent imaging on 14 d. The miR-497 expression level of tumor tissues was confirmed by real-time PCR assay. Compared with the control, the expression level of miR-497 significantly increased in miR-497 mimic group (*P *< 0.05) (Fig. 4A). To evaluate the pro-apoptosis role of miR-497 via targeting VEGFR2 and its downstream proteins *in vivo*, western blotting was performed to detect the expression of VEGFR2, Bax and Bcl-2. The data showed that compared with control, VEGFR2 and Bcl-2 expression in miR-497 mimic group was significant lower, while pro-apoptosis protein Bax was higher (*P *< 0.05) (Fig. 4B–D).

The regulating role of miR-497 in tumor growth was further investigated by TUNEL analysis, which displayed that only few TUNEL positive cells was in the control group, while a significant increase of TUNEL-positive cells was observed in miR-497 mimic group (*P *< 0.05) (Fig. 4E,F). However, the tumors transfected with miR-497 mimic-NC did not produce noticeable changes as compared to control (*P *> 0.05) (Fig. 4E,F). Taken together, the up-regulation of miR-497 inhibits tumor growth by inducing cell apoptosis.

### Overexpression of miR-497 inhibits tumor angiogenesis *in vivo*

**Figure 5:**
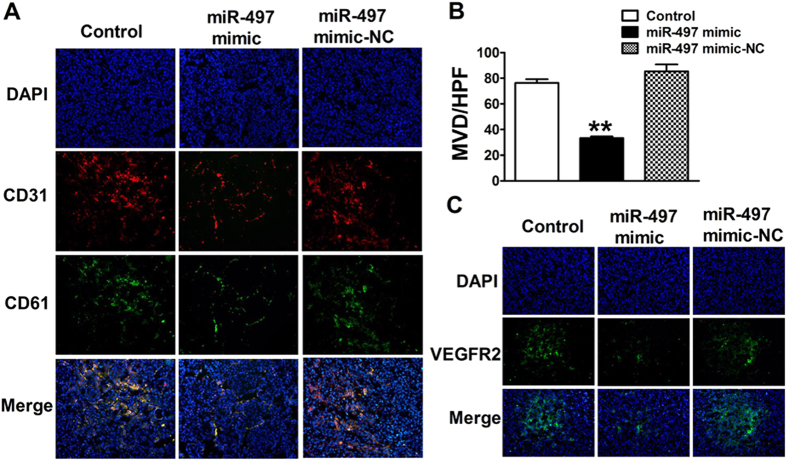
Representative CD31, CD61 and VEGFR2 immunostaining images were carried out under different experimental conditions as indicated (Original magnification, ×200). (**A**) CD31 was stained with red color and CD61 was stained with green color; (**B**) MVD of tumors in each group was detected by the vessel counts. MVD: micro-vessel density, HPF: high-power field. Data were expressed as mean ± SEM, n = 10; ^**^P < 0.01 *vs*. control group; (**C**) VEGFR2 was stained with green color, and cells nuclei were blue.

In this study, immunofluorescent staining with CD31 and CD61 was applied to testify MVD in the tumor sections. The results demonstrated that the mean MVD of control, miR-497 mimic and miR-497 mimic-NC groups was 76 ± 1.6, 35 ± 1.2 and 82 ± 3.5, respectively. The tumor sample from miR-497 mimic group had less capillary blood vessels than the control group (35 ± 1.2 *vs*. 76 ± 1.6, *P *< 0.05) (Fig. 5A,B), while compared with the control, MVD of tumor section in miR-497 mimic-NC group showed no statistical difference (82 ± 3.5 *vs*. 76 ± 1.6, *P *> 0.05) (Fig. 5A,B). More importantly, the negative correlation between miR-497 expression and MVD of tumor tissues was found in this study, which suggested that the miR-497 played a vital role in suppressing tumor angiogenesis.

To further investigate the effect of miR-497 on tumor angiogenesis in breast tumor models, tumor sections were also determined by immunofluorescent staining assay of VEGFR2. We monitored the expression of VEGFR2 which was closely associated with tumor angiogenesis in each group. Compared with the control, tumor tissue sections from the miR-497 mimic group exhibited lower grade of VEGFR2 positive staining (Fig. 5C), which was consistent with the results of bioluminescent imaging *in vivo*. In contrast, the grade of VEGFR2 positive stained in miR-497 mimic-NC group did not significantly change compared with control (Fig. 5C).

**Figure 6:**
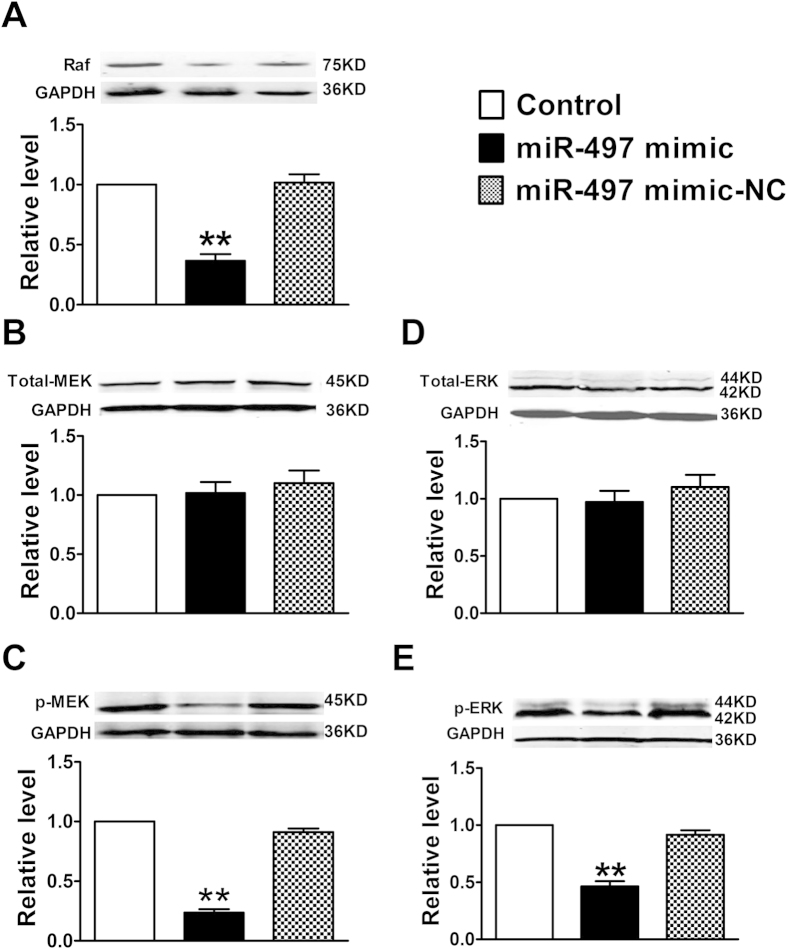
Effects of miR-497 overexpression on Raf/MEK/ERK signaling pathway in mouse breast tumor model. Proteins expression of Raf, Total-MEK, p-MEK, Total-ERK and p-ERK were determined by western blotting assay. Data were expressed as mean ± SEM, n = 3; ^**^P < 0.01 *vs*. control group.

Next, in order to elucidate the anti-angiogenesis effects of miR-497 mediated by down-regulation of VEGFR2/Raf/ERK/MEK pathway in breast tumor, we investigated the expression of angiogenesis-related signal pathway proteins in tumor lysates from these three groups. Western blotting analysis displayed that overexpression of miR-497 could down-regulate the endogenous levels of Raf, p-ERK and p-MEK *in vivo* (*P *> 0.05) (Fig. 6), meanwhile the expression of total ERK and MEK did not change compared to control (*P *> 0.05) (Fig. 6). The above results confirmed that miR-497 could markedly inhibit breast tumor angiogenesis *in vivo*.

## Discussion

**Figure 7:**
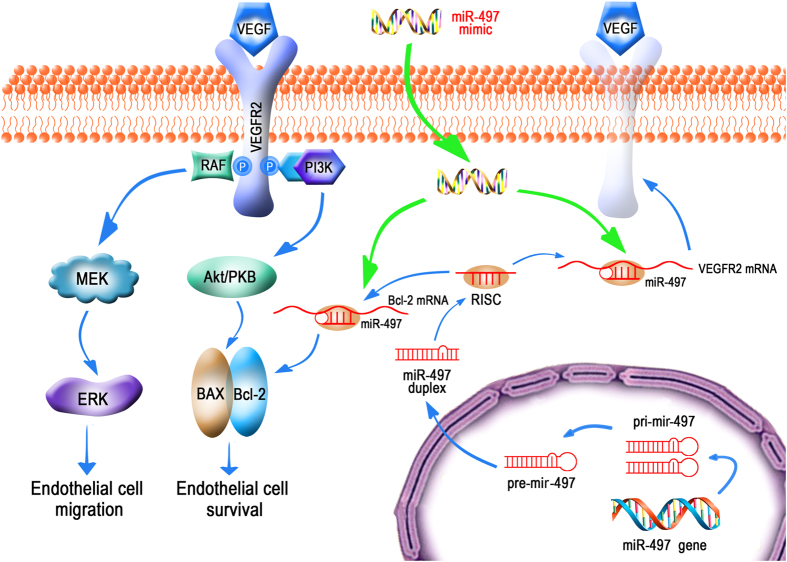
Schematic illustrations explain the possible targeting and signalling mechanisms by which miR-497 produces anti-angiogenesis effects in *in vitro* and *in vivo* model. Increased miR-497 represses angiogenesis by targeting VEGFR2, which lead to decreases in the activation of both PI3K/AKT and Raf/MEK/ERK signaling pathways. In addition, miR-497 may induce endothelial cells apoptosis by directly down-regulating anti-apoptotic factor Bcl-2 expression. And this schematic diagram was drawn by Yingfeng Tu and Xiaowei Ma.

In this study, several novel findings have been disclosed. Firstly, we demonstrated, for the first time, that VEGFR2 was the target gene of miR-497, which could be down-regulated by overexpression of miR-497 in HUVECs and 4T1 breast cancer cells. Secondly, as shown in Fig. 7, up-regulation of miR-497 level was able to induce HUVECs apoptosis via targeting VEGFR2/PI3K/AKT signaling pathway and trigger anti-proliferative action through VEGFR2/Raf/ERK/MEK signaling pathway in HUVECs. Thirdly, overexpression of miR-497 produced anti-tumor and anti-angiogenesis effects in breast tumor-bearing mice, and VEGFR2-luc mouse was a useful tool in monitoring the therapeutic anti-angiogenesis effect of miR-497 *in vivo*. In brief, these results not only help us to comprehend the mechanisms underlying the anti-angiogenesis effects of miR-497 but also improve our view of miRNAs that may serve as potential therapeutic and drug targets in future.

Increasing evidence suggests that miRNAs, which are non-coding small RNAs, may act as oncogenes or tumor suppressor genes^18^. Si *et al*. demonstrated that the suppression of miR-21 increased apoptosis both *in vitro* and in the xenograft mouse model via the down-regulation of Bcl-2^32^. Nicoli *et al*. reported that miR-126 plays an essential role in angiogenesis by modulating VEGFR2-related signal transduction through the RAS/ERK and PI3K/AKT pathways by inhibiting regulatory units^33^. Moreover, the previous studies provided information that miR-497 had effects on inhibiting cell viability and inducing apoptosis in several tumor cell lines. For instance, Liu and co-workers found that has-miR-497 could play a role in both gastric and lung cancer cell lines, at least in part modulating apoptosis via targeting Bcl-2 28. Shen *et al*. reported that miR-497 induced apoptosis of breast cancer cells by acting on Bcl-w^31^. Zhang *et al*. identified down-regulation of miR-497 as an important mechanism of up-regulation of IGF1-R in colorectal cancer cells, which contributed to malignancy of colorectal cancer^29^. Creevey *et al*. verified that overexpression of miR-497 reduced cell viability and increased apoptosis in MNA cells at the same time, which could be used as a potential therapeutic agent for high-risk neuroblastoma^34^. Recently, Zhao *et al*. found that ectopic expression of miR-497 *in vivo* significantly inhibited tumor growth and tumor angiogenesis in severe combined immune deficiency (SCID) mouse xenograft model of non-small cell lung cancer^35^. However, the direct role of miR-497 in regulating tumor angiogenesis has not yet been fully disclosed.

Our current study presented the first evidence that up-regulation of miR-497 greatly induced HUVECs apoptosis and inhibited HUVECs growth via targeting VEGFR2 and its downstream signaling pathway proteins, which provided the direct evidence that miR-497 was able to modulate tumor angiogenesis (summarized in Fig. 7). PI3K/Akt pathway is well known to be a major cell survival pathway, and the activation of the PI3K/Akt pathway enhances resistance to cell apoptosis^36^,^37^. The results in this study demonstrated that up-regulation of miR-497 could markedly induce HUVECs apoptosis through inhibiting PI3K/Akt signaling pathway by targeting upstream regulator VEGFR2. In addition, we also demonstrated that overexpression of miR-497 could induce HUVECs apoptosis by targeting Bcl-2 (see Supplementary material online, Fig. S3), which was in line with previous studies^27^. Next, we investigated the underlying mechanism and relative signaling molecules of miR-497 in the anti-proliferative action in HUVECs. In accord with previous study^30^, we also found that overexpression of miR-497 led to the inhibition of Raf/MEK/ERK signaling pathway, suggesting miR-497 was involved in modulating proliferation in HUVECs *in vitro*.

Anti-angiogenic therapy is one of important methods in treating tumors. In recent studies, the detection of newborn tumor blood vessels and monitoring of anti-angiogenic drugs effects are being widely studied^38^,^39^. However, till now, reliable methods of measuring angiogenesis *in vivo* are required. Traditional biological techniques, like western blotting and immunohistochemical method, usually require euthanasia of experimental animals to acquire the relevant biological information. Therefore, the dynamic observations of the anti-angiogenic response could not be continuously carried out in the same animal over time. In recent years, with the development of optical molecular imaging technique, such as BLI, we can monitor the effects of anti-angiogenic drugs at the molecular level *in vivo* and evaluate therapeutic efficacy much earlier and more accurately^40^. In contrast with traditional biological technology, BLI can noninvasively visualize the biological process at cellular or molecular levels in an intact system in the same animal through a longitudinal study, provided that more statistically correlative and more accurate results are presented in evaluating the efficacy of antitumor drugs. Therefore, in this study, we furthermore investigated if the beneficial effects of miR-497 exist in *in vivo* conditions. In order to disclose the relationship between miR-497 and tumor angiogenesis, VEGFR2-luc transgenic mice were applied to repetitively and non-invasively monitor tumor growth and angiogenesis by using BLI. In line with what we expected, overexpression of miR-497 could significantly inhibit breast tumor growth and produced ant-angiogenesis effect *in vivo*. Concomitantly, the expression of VEGFR2 could be successfully suppressed by miR-497 overexpression *in vivo*, which was consistent with the *in vitro* results. In addition, the immunostaining analysis further demonstrated that miR-497 was involved in inhibiting tumor angiogenesis in mouse tumor model.

In conclusion, our study indicates that VEGFR2, which is required for inducing angiogenesis, is a key downstream target of miR-497. *In vitro* overexpression of miR-497 is able to induce HUVECs apoptosis and inhibit cell proliferation via targeting VEGFR2/PI3K/AKT and VEGFR2/Raf/MEK/ERK signaling pathways respectively, which play important role in regulating miR-497-mediated anti-angiogenesis role. And *in vivo* BLI data show that miR-497 inhibits tumor angiogenesis via targeting VEGFR2 and its downstream signaling pathways, suggesting that miR-497 may be a promising intervention in the management of tumor angiogenesis and a potential drug candidate for cancer therapy in future.

## Methods and Materials

### Cell culture

The breast cancer cell line 4T1 was purchased from American Type Culture Collection (ATCC, USA). 4T1 breast cancer cells were maintained in RPMI 1640 medium supplemented with 10% fetal bovine serum (FBS) (Invitrogen, USA) and 50 IU penicillin/streptomycin. Human umbilical vein endothelial cells (HUVECs) were also purchased from ATCC and cultured in Fisher M199 medium (Invitrogen, USA) supplemented with 20% FBS, 1% penicillin/streptomycin, 100 μg/mL Heparin (Sigma), and 50 μg/mL Endothelial Cell Growth Supplement (ECGS) (BD Sciences). All cells were cultured at 37 °C in an incubator containing 5% CO_2_.

### Animals

In 2004, Zhang and his colleagues utilized a transgene to create original VEGFR2-luc mice, which comprised of a murine VEGFR2 promoter region cloned upstream from the luciferase gene^41^. Because luciferase would be transcribed when VEGFR2 was transcriptionally activated, VEGFR2 expression of this transgenic mouse could be non-invasively and quantitatively monitored in real-time using BLI. In this study, we used the VEGFR2-luc mice generated in FVB/N mouse strain to establish breast tumor model, which were purchased from Caliper Life Sciences Inc. (Hopkinton, USA). In accordance with the National Institutes of Health guidelines, mice were housed under pathogen-free conditions. All *in vivo* experiment protocols were approved by the Animal Care and Use Committee at the Harbin Medical University.

### Transfection of miR-497 mimic or inhibitor into cultured HUVECs

The cells were seeded in antibiotic-free medium for 24 h prior to transfection. For the miR-497 up-regulation, the cells were transfected with miR-497 mimic or mimic control (GenePharma Co. Ltd.) using Lipofectamine 2000 (Invitrogen, USA). Transfection complexes were added to medium at final oligonucleotide concentration of 50 nM. In the same way, the HUVECs were transfected with miR-497 inhibitor or inhibitor control at final oligonucleotide concentration of 50 nM to knockdown miR-497. The culture medium was replaced 4 h post-transfection with the regular culture medium for 24 h. miRNA transfection efficiency was proved by qRT-PCR.

### MTT Proliferation Assay

For cells proliferation viability assay, a modified MTT assay was used to examine the cell proliferation. Briefly, HUVECs (50 ~ 60% density) transfected with 100 nM miR-497 mimic, miR-497 mimic-NC, miR-497 inhibitor or inhibitor-NC were seeded in 96-well plates and incubated at 37 °C for 48 h. Cells transfected nothing were used as control. Then 20 μL of MTT dye solution was added to each well and incubated for additional 4 h. Subsequently, the supernatant was discarded and 150 μL/well DMSO was added to dissolve formazan crystals. Absorbance values were determined at 570 nm on a 96-well microplate reader. All MTT assays were repeated three times.

### Dual-luciferase reporter assay

HUVECs were plated at 2 × 10^5^ cells per well in 24-well plates. The following day, cells were co-transfected with 80 ng of pMIR-REPORT Luciferase vector, including the 3′UTR of VEGFR2 (with either wild-type or mutant miR-497 binding sites), pRL-TK control vector (encoding Renilla luciferase, 8 ng), and miR-497 mimic or mimic control at a final concentration of 50 nm by using Lipofectamine 2000 (Invitrogen) according to the manufacturer’s instructions. After transfection for 48 hours, firefly and renilla luciferase activities were performed by using the Dual-Luciferase Reporter Assay (Promega, USA). Normalized data were calculated as the quotient of Renilla/firefly luciferase activities. Each experiment was repeated for at least three times in each group.

### Established tumor model and treatment protocol *in vivo*

Approximately 2 × 10^6^ 4T1 breast cancer cells suspended in 100 μL of phosphate buffered saline (PBS) were implanted subcutaneously beneath the fat pad of mice, which were anesthetized with 2.5% isofluorane. A tumor bulb could be seen seven days after the tumor cells were injected and tumor sizes were assessed by caliper measurements. Mice were randomized into three groups with the very similar mean tumor volume: control group, miR-497 mimic group, miR-497 mimic-NC group (n = 5 in each group). Animals received intratumoral injections of miR-497 mimic or miR-497 mimic-NC respectively for 1 dose (50 mg/kg mimic or inhibitor dissolved in 100 μL mixed solution) at day 0. The same volume of saline was locally injected into the tumor mass as control. For each tumor, five intratumoral injection sites were used to make sure the drug diffusion evenly. Two perpendicular tumor diameters (*a* stands for the longest diameter and *b* stands for the shortest diameter) were measured every day to observe tumor progression. Tumor volumes were calculated as volume = π*ab*^2^/6.

### Bioluminescent imaging

*In vivo* BLI was conducted on the IVIS 200 system with the use of the Living Image acquisition and analysis software (Caliper Life Sciences). Before imaging, the mice were anesthetized with 2.5% isofluorane and the luciferase substrate D-luciferin firefly (Caliper Life Sciences) at the dose of 150 mg/kg was administered by intraperitoneal injection. Serial imaging of the tumor area was done between 10 and 20 min after injection of luciferin to capture the peak intensity, which could well represent tumor volume. After separated mice into three groups, BLI was performed on 0 d (treatment day), 3 d, 5 d, 7 d and 14 d. Sum of all detected photon counts within an oval shaped region of interest (ROI) was quantified in units of mean photons per second per centimeter squared per steradian (P/s/cm^2^/sr) as described previously using Living Image® software .

### Immunofluorescence analysis

Mice were sacrificed by fatal overdose of anesthesia, and tumors of different experiment groups were removed and fixed in cryopreservation medium. Breast tumors were sliced in 5 mm sections and mounted on glass slides for immunofluorescence experiments. Slices were firstly fixed with ice-cold acetone for 10 min. After drying at room temperature, sections were then blocked with 10% goat serum for 30 min at room temperature, and mouse anti-CD31, anti-CD61 monoclonal antibody (Abcam) or rabbit anti-VEGFR2 polyclonal antibody (Cell Signaling Technology) were used as primary antibodies for overnight incubation at 4 °C. The sections were subsequently treated with goat anti-mouse IgG-Alexa Fluor 594 (1:500 dilution, Invitrogen) or goat anti-mouse IgG-Alexa Fluor 488 (1:500 dilution, Invitrogen) for 1 h. Finally, cell nuclei were stained with DAPI. Image of tumor tissue was captured with fluorescence microscope (IX71+DP72, Olympus). All pictures were taken under 200× magnification with the same exposure time.

### Quantification of micro-vessel density

For angiogenesis quantification, micro-vessel density (MVD) was assessed with CD31 and CD61 double staining. Briefly, areas of containing the highest number of capillaries were identified by scanning the tumor sections at low power, and vessels both labeled with CD31 and CD61 were assessed by counting in 10 random fields at 200×magnification under the fluorescence microscope. In order to determine the mean number of micro-vessel density within the tumor, mean MVD was calculated by the average number from ten randomized fields. All quantitative evaluations were carried out by ImagePro Plus software (version 6.0, Media Cybernetics).

### Assessment of apoptosis

Apoptosis of HUVECs, 4T1 breast cancer cells and tumor tissues in different experiment group was performed using TUNEL assay according to manufacturer’s instruction. TUNEL staining was done using the *in situ* cell death detection kit (Roche). Then HUVECs and tumor tissue slice were observed under microscope and positive cells were counted in 10 high magnification fields (HPF). Apoptotic index was determined as the percentage of TUNEL-positive cells and expressed as positive cells/HPF. Images were captured with fluorescence microscope (IX71 + DP72, Olympus) and cell apoptosis was determined with ImagePro Plus software.

### Quantitative reverse transcription-PCR (qRT-PCR)

Total RNA was isolated from HUVECs, 4T1 breast cancer cells and tumor tissues in each group using Trizol (Invitrogen, USA) according to the manufacturer’s instructions. Then real-time PCR was used to quantify the expression of miR-497 with specific Taqman assays (Applied Biosystems, USA) and Taqman universal master mix (Applied Biosystems, USA). cDNA was obtained from total RNA using High-Capacity cDNA Reverse Transcription Kit (Applied Biosystems, USA). Real-time PCR was performed using the SYBR Green PCR Master Mix Kit (Applied Biosystems, USA) on a 7500 Fast Real-Time PCR System (Applied Biosystems, USA) to quantify the level of miR-497. U6 was used as an internal control. The primers were designed as follows: miR-497 forward, 5′-GTGCAGGGTCCGAGGT-3′; miR-497 reverse, 5′-TAGCCTGCAGCACACTGTGGT-3′; U6 forward, 5′-GCTTCG GCACATATACTAAAAT-3′; U6 reverse 5′-CGCTTCACGAATTTGCGTGTCAT-3′.

### Western blot analysis

The total amount of protein was extracted from HUVECs, 4T1 breast cancer cells and tumor tissues in each group for immunoblotting analysis. Briefly, the protein concentrations were determined with a bicinchoninic acid protein assay kit using bovine serum albumin as the standard. Equal amounts of protein (100 μg) were fractionated by SDS-PAGE and blotted to PVDF membrane (Millipore, Bedford, MA). The PVDF membrane was blocked with 5% non-fat milk and incubated with the primary antibody at 4 °C overnight. Then the blots were incubated with secondary antibody: Alexa Fluor® 800 goat anti-mouse or anti-rabbit IgG (Invitrogen) for 1 h at room temperature. The primary antibodies against VEGFR2, Akt, phosphorylated of Akt (p-Akt), Bcl-2, Bax, MEK, p-MEK, ERK, p-ERK, Raf and GAPDH were purchased from Abcam and Cell Signaling. Western blot bands were captured by using the Odyssey Infrared Imaging System (LI-COR Biosciences) and quantified with Odyssey v1.2 software (LI-COR Biosciences). GAPDH was used as the internal control. Western blotting experiments were repeated four times.

### Statistical Analysis

All quantitative data were expressed as the mean ± standard error of the mean (SEM). Statistical analyses were performed using the Student’s *t* test for comparisons of two groups and using one-way ANOVA for multi-group comparisons. Significance was set at *P *< 0.05.

## Additional Information

**How to cite this article**: Tu, Y. *et al*. Overexpression of miRNA-497 inhibits tumor angiogenesis by targeting VEGFR2. *Sci. Rep*. **5**, 13827; doi: 10.1038/srep13827 (2015).

## Supplementary Material

Supplementary Information

Supplementary Figures
