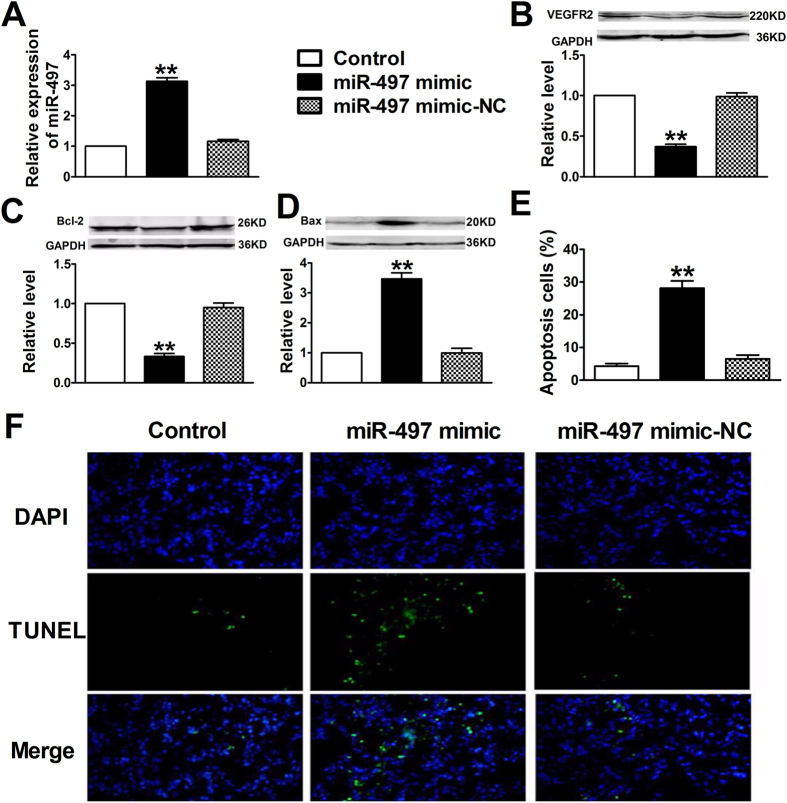# Corrigendum: Overexpression of miRNA-497 inhibits tumor angiogenesis by targeting VEGFR2

**DOI:** 10.1038/srep21221

**Published:** 2016-02-16

**Authors:** Yingfeng Tu, Li Liu, Dongliang Zhao, Youbin Liu, Xiaowei Ma, Yuhua Fan, Lin Wan, Tao Huang, Zhen Cheng, Baozhong Shen

Scientific Reports 5: Article number: 13827; 10.1038/srep13827 published online: 09082015; updated: 10162015.

In this Article, Figure 4 is incorrect. The correct Figure 4 appears below as Figure 1.

## Figures and Tables

**Figure 1:**