# Supplementary material for: Overexpression of miRNA-497 inhibits tumor angiogenesis by targeting VEGFR2
Source: Sci Rep. 2015 Sep 8;5:13827. doi: 10.1038/srep13827 (PMC4561885; doi:10.1038/srep13827)
Supplement: Supplementary Information [file srep13827-s1.doc]

**Overexpression of miRNA-497 inhibits tumor angiogenesis by targeting VEGFR2**

Yingfeng Tu^1,2,5§^, Li Liu^3§^, Dongliang Zhao^1,4^, Youbin Liu^5^, Xiaowei Ma^6^, Yuhua Fan^7^, Lin Wan^1,4^, Tao Huang^1,4^, Zhen Cheng^6*^, Baozhong Shen^1,4*^

^1^Key Laboratory of Molecular Imaging, College of Heilongjiang Province, Harbin, Heilongjiang, China

^2^Department of Cardiology, the Fourth Hospital of Harbin Medical University, Harbin, Heilongjiang, China

^3^Department of Anesthesiology, the Third Hospital of Harbin Medical University, Harbin, Heilongjiang, China

^4^Department of Radiology, the Fourth Hospital of Harbin Medical University, Harbin, Heilongjiang, China

^5^Department of Cardiology, the Second Hospital of Harbin Medical University, Harbin, Heilongjiang, China

^6^Molecular Imaging Program at Stanford, Department of Radiology and Bio-X Program, Stanford University, Stanford, California, USA

^7^College of Pharmacy, Harbin Medical University, Daqing, Heilongjiang, China

§These authors contributed equally to this work.

*Correspondence should be sent to:

Baozhong Shen, M.D., Ph.D.

Key Laboratory of Molecular Imaging, College of Heilongjiang Province

Department of Radiology, the Fourth Hospital of Harbin Medical University,

Harbin, Heilongjiang, China, 150001

Email: [shenbzh@vip.sina.com](mailto:shenbzh@vip.sina.com)

Or

Zhen Cheng, Ph.D.
Molecular Imaging Program at Stanford

Canary Center at Stanford for Cancer Early Detection
Department of Radiology and Bio-X Program

Stanford University, Stanford, CA 94305

E-mail: [zcheng@stanford.edu](mailto:zcheng@stanford.edu)

**Material and Methods**

**Dual-luciferase reporter assay**

HUVECs were plated at 2×10^5^ cells per well in 24-well plates. The following day, cells were co-transfected with 80 ng of pMIR-REPORT Luciferase vector, including the 3′UTR of Bcl-2 (with either wild-type or mutant miR-497 binding sites), pRL-TK control vector (encoding Renilla luciferase, 8 ng), and miR-497 mimic or mimic control at a final concentration of 50 nm by using Lipofectamine 2000 (Invitrogen) according to the manufacturer’s instructions. After transfection for 48 hours, firefly and renilla luciferase activities were performed by using the Dual-Luciferase Reporter Assay (Promega, USA). Normalized data were calculated as the quotient of Renilla/firefly luciferase activities. Each experiment was repeated for at least three times in each group.

**Supplemental Figure Legends**

**Supplementary Figure 1.** VEGFR2 was validated as a target of miR-497 in HUVECs. (A) 3’ UTR of VEGFR2 harbored a potential targeting site of miR-497, which was conserved among mouse, human, dog and cat, by bioinformatics analysis. (B-C) Luciferase assay was performed to show that overexpression of miR-497 in HUVECs could significantly suppress the luciferase activity of a reporter fused with 3′ untranslated region of VEGFR2 mRNA. HUVECs were transfected with a pMIR-VEGFR2-3′-UTR or pMIR-VEGFR2-m3′-UTR, respectively. Meanwhile, the cells were co-transfected with a miR-497 mimic or mimic control. Compared with the mimic control, the miR-497 mimic could reduce luciferase activity containing a wild-type miR-497 binding site (*P* <0.01 *vs.* mimic control) (B) but not a mutant binding site (C).

**Supplementary Figure 2.** Effects of expression level of miR-497 on cultured HUVECs survival. Cell apoptosis was determined by TUNEL staining. (A) Apoptotic cells were stained with green color by TUNEL; cell nuclei were stained with blue color by DAPI. Images were acquired at a magnification of ×200. (B) Number of cell apoptosis was calculated from conditions in A and expressed in percentage. Data were expressed as mean±SEM, n=10; *^***^P*<0.001 *vs.* control group.

**Supplementary Figure 3.** Bcl-2 was validated as a target of miR-497 in HUVECs. (A) 3’ UTR of Bcl-2 harbored a potential targeting site of miR-497, which was conserved among mouse, human, dog and cat, by bioinformatics analysis. (B) Luciferase assay was performed to show that overexpression of miR-497 in HUVECs could significantly suppress the luciferase activity of a reporter fused with 3′ untranslated region of Bcl-2 mRNA. HUVECs were transfected with a pMIR-Bcl-2-3′-UTR or pMIR-Bcl-2-m3′-UTR, respectively. Meanwhile, the cells were co-transfected with a miR-497 mimic or mimic control. Compared with the mimic control, the miR-497 mimic could reduce luciferase activity containing a wild-type miR-497 binding site (*P* <0.01 *vs.* mimic control) but not a mutant binding site.

**Supplementary Figure 4.** Effects of expression level of miR-497 on cultured 4T1 cells survival. (A) Quantitative analysis of miR-497 expression in cultured 4T1 cells by qRT-PCR analysis; (B-D) Proteins expression of VEGFR2, Bcl-2, and Bax. Data were expressed as mean±SEM, n=3; ^**^*P*<0.01 *vs.* control group. (E) Apoptotic cells were stained with green color by TUNEL; cell nuclei were stained with blue color by DAPI. Images were acquired at a magnification of ×200. (F) Number of cell apoptosis was calculated from conditions in A and expressed in percentage. Data were expressed as mean±SEM, n=10; *^**^P*<0.01 *vs.* control group.


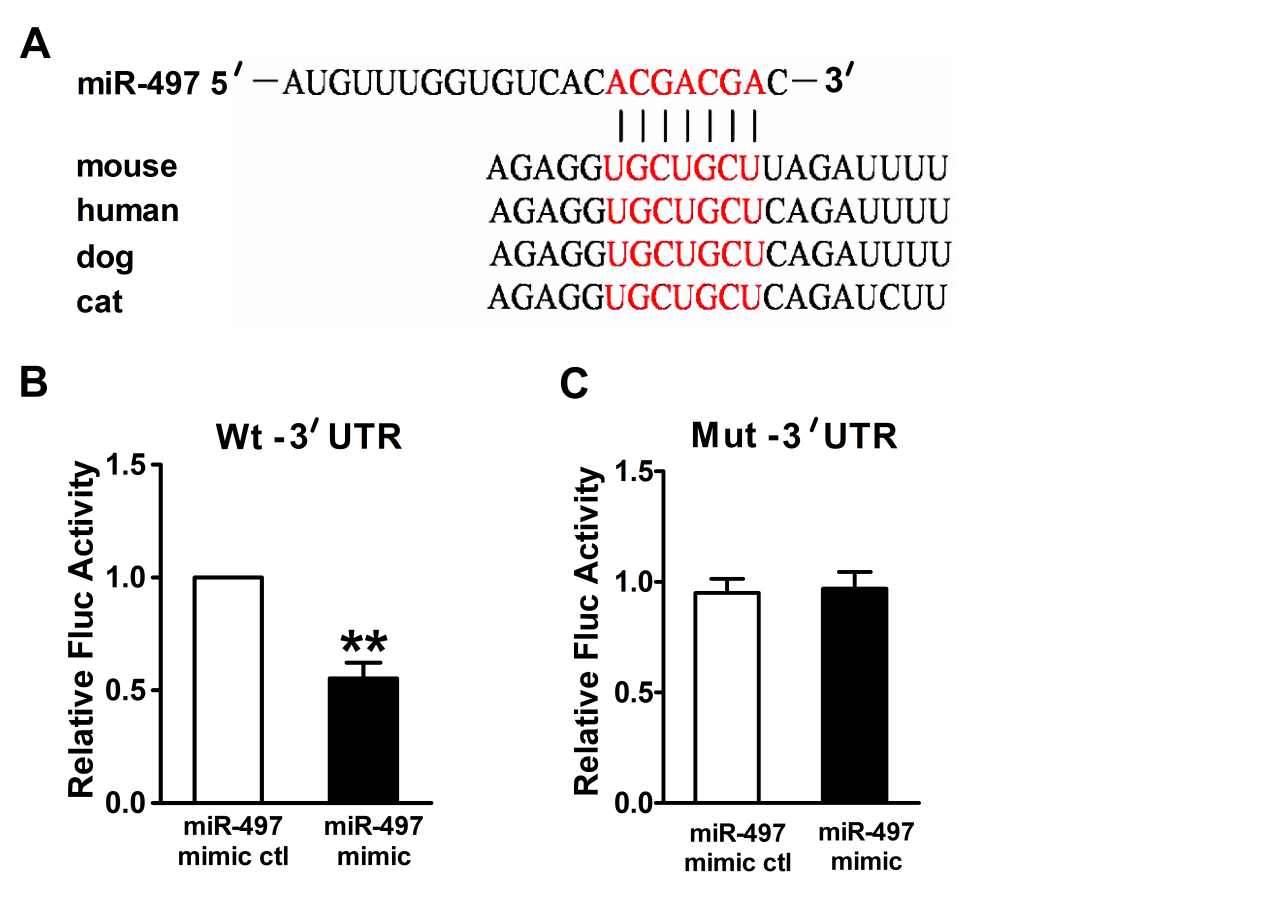


Supplement Fig. S1


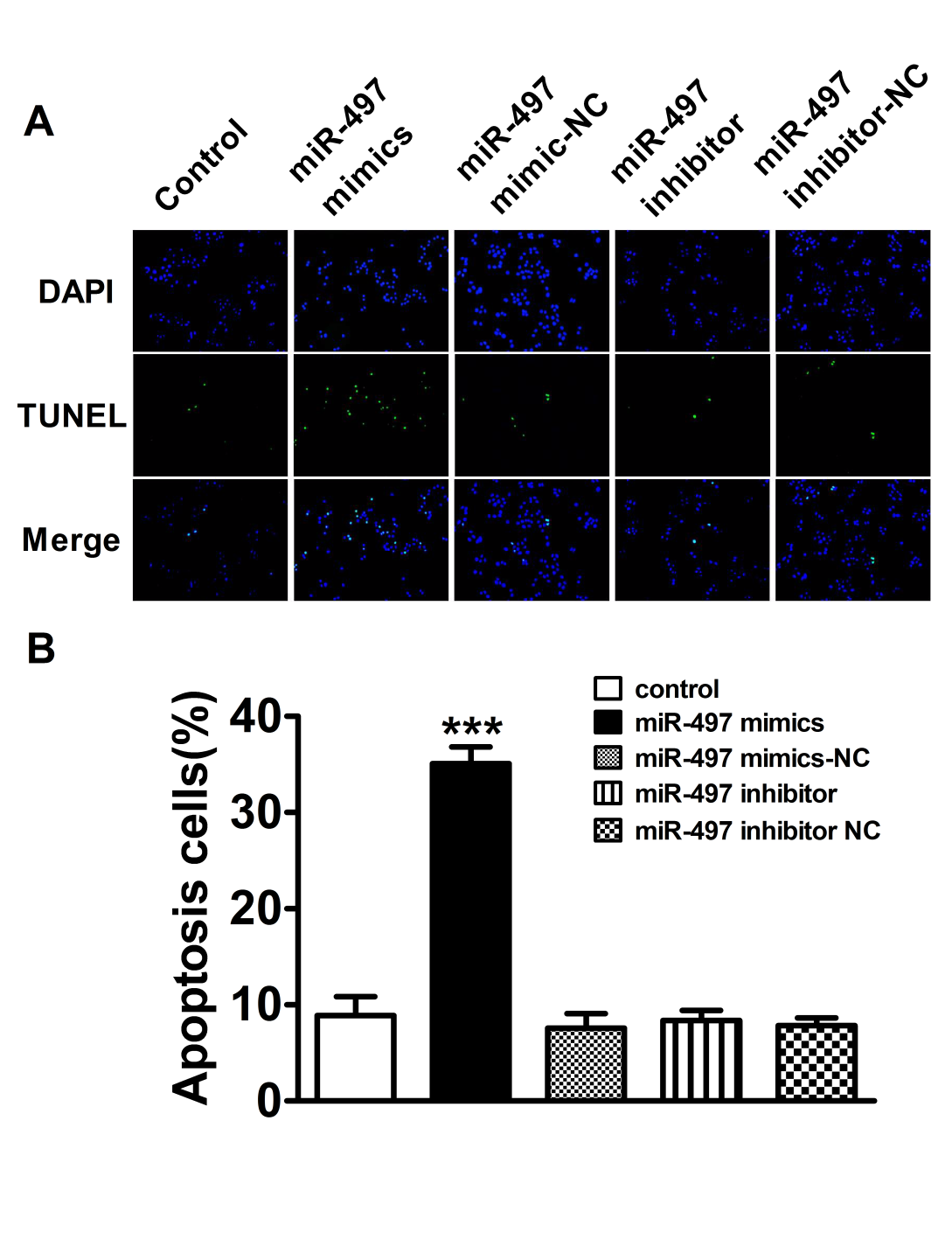


Supplement Fig. S2


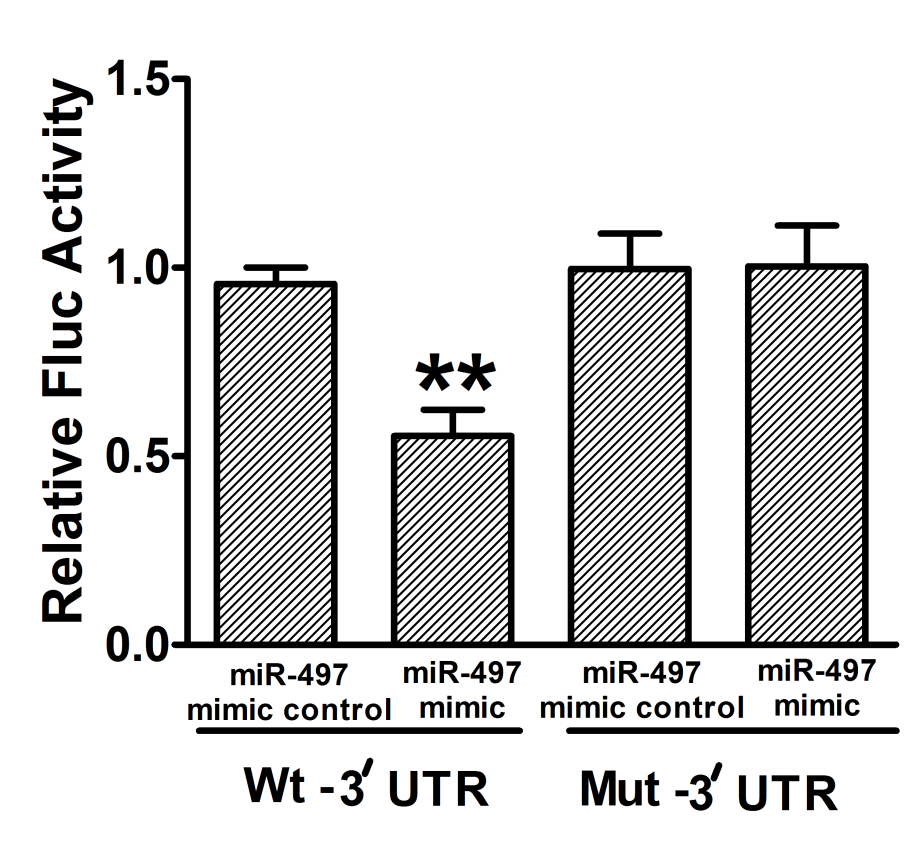


Supplement Fig. S3


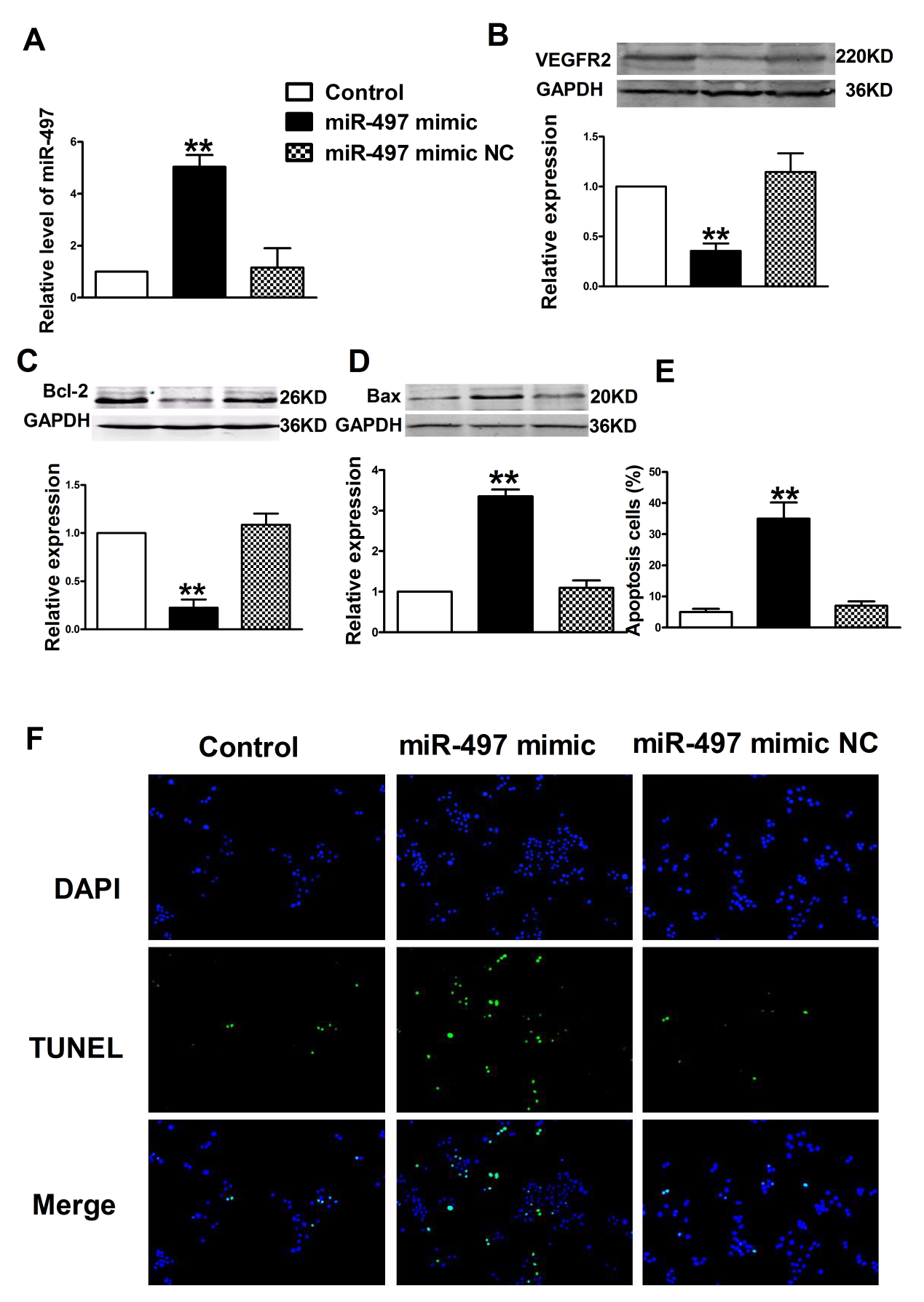


Supplement Fig. S4
