# Supplementary material for: Overexpression of miRNA-497 inhibits tumor angiogenesis by targeting VEGFR2
Source: Sci Rep. 2015 Sep 8;5:13827. doi: 10.1038/srep13827 (PMC4561885; doi:10.1038/srep13827)
Supplement: Supplementary Figures [file srep13827-s2.doc]

**Overexpression of miRNA-497 inhibits tumor angiogenesis by targeting VEGFR2**

Yingfeng Tu^1,2,5§^, Li Liu^3§^, Dongliang Zhao^1,4^, Youbin Liu^5^, Xiaowei Ma^6^, Yuhua Fan^7^, Lin Wan^1,4^, Tao Huang^1,4^, Zhen Cheng^6*^, Baozhong Shen^1,4*^

^1^Key Laboratory of Molecular Imaging, College of Heilongjiang Province, Harbin, Heilongjiang, China

^2^Department of Cardiology, the Fourth Hospital of Harbin Medical University, Harbin, Heilongjiang, China

^3^Department of Anesthesiology, the Third Hospital of Harbin Medical University, Harbin, Heilongjiang, China

^4^Department of Radiology, the Fourth Hospital of Harbin Medical University, Harbin, Heilongjiang, China

^5^Department of Cardiology, the Second Hospital of Harbin Medical University, Harbin, Heilongjiang, China

^6^Molecular Imaging Program at Stanford, Department of Radiology and Bio-X Program, Stanford University, Stanford, California, USA

^7^College of Pharmacy, Harbin Medical University, Daqing, Heilongjiang, China

§These authors contributed equally to this work.

*Correspondence should be sent to:

Baozhong Shen, M.D., Ph.D.

Key Laboratory of Molecular Imaging, College of Heilongjiang Province

Department of Radiology, the Fourth Hospital of Harbin Medical University,

Harbin, Heilongjiang, China, 150001

Email: [shenbzh@vip.sina.com](mailto:shenbzh@vip.sina.com)

Or

Zhen Cheng, Ph.D.
Molecular Imaging Program at Stanford

Canary Center at Stanford for Cancer Early Detection
Department of Radiology and Bio-X Program

Stanford University, Stanford, CA 94305

E-mail: [zcheng@stanford.edu](mailto:zcheng@stanford.edu)

**Figure 1B (VEGFR2)**


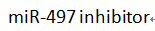

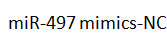

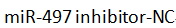


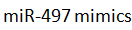


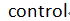


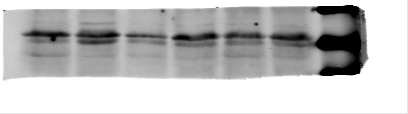


**210KD**

**170KDD**

**250KD**

**VEGFR2**

**220KD**

**220KD**

**Figure 1C (Total Akt)**


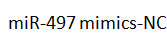

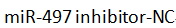

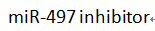

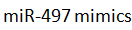

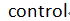


**95KD**


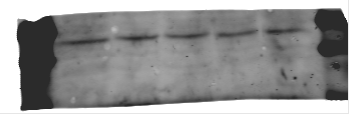


**55KD**

**72KD**

**Total Akt**

**60KD**

**Figure 1D ( p-Akt)**


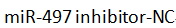


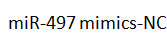

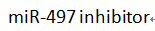

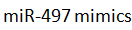


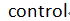


**72KD**


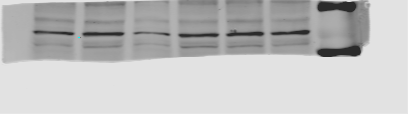


**p-Akt**

**60KD**

**55KD**

**Figure 1E (Bax)**

**
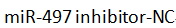

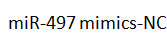
**

**
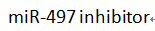

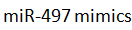
**

**
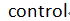
**

**26KD**

**
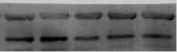
**

**Bax**

**20KD**

**17KD**

**Figure 1F (Bcl-2)**


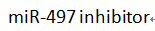

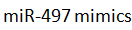

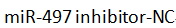

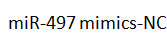


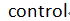


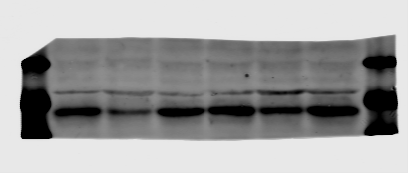


**26KD**

**17KD**

**34KD**

**Bcl-2**

**26KD**

**Figure 2B (Raf)**


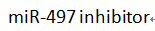

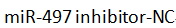

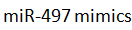

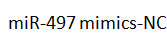


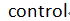


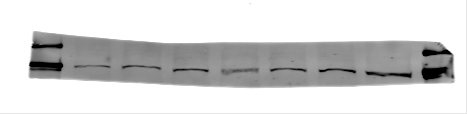


**95KD**

**72KD**

**Raf**

**75KD**

**Figure 2C (Total MEK)**

**
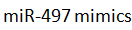

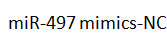

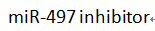

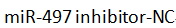

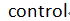
**


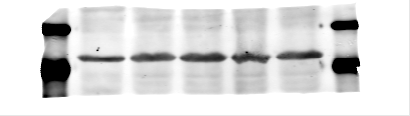


**34KD**

**55KD**

**Total-MEK**

**45KD**

**Figure 2D ( p-MEK)**


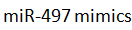

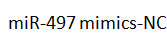

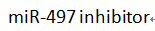

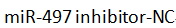

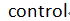


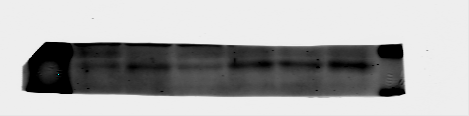


**34KD**

**55KD**

**p-MEK**

**45KD**

**Figure 2E (Total ERK)**

**
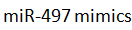

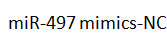

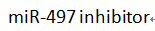

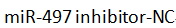

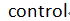
**


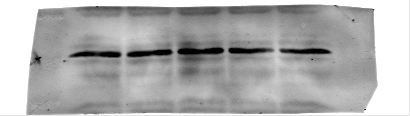


**Total-ERK**

**44KD**

**42KD**

**55KD**

**34KD**

**26KD**

**Figure 2F (p-ERK)**

**
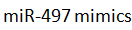

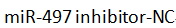

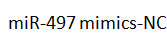

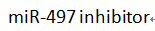
**

**
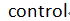
**

**
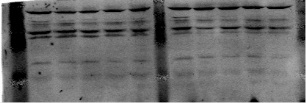
**

**34KD**

**55KD**

**72KD**

**Total-ERK**

**44KD**

**42KD**

**Figure 4B (Raf)**

**
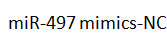
**

**
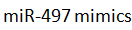

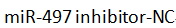

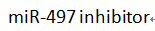
**

**
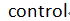
**

**Raf**

**75KD**

**95KD**

**
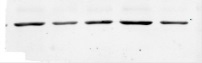
**

**55KD**

**72KD**

**Figure 4C (Total MEK)**

**
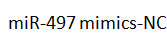
**

**
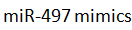

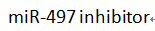

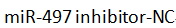
**


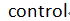


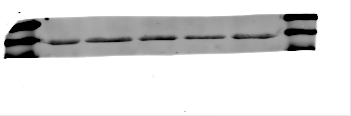


**34KD**

**55KD**

**Total-MEK**

**45KD**

**26KD**

**Figure 4D (p-MEK)**

**
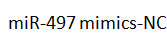
**

**
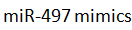

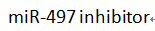

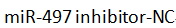
**

**
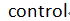
**

**
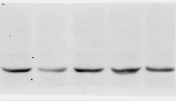
**

**p-MEK**

**45KD**

**34KD**

**55KD**

**72KD**

**Figure 4E (Total ERK)**

**
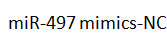

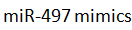

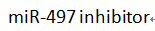

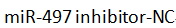
**


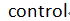


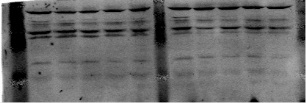


**Total-ERK**

**44KD**

**42KD**

**34KD**

**55KD**

**72KD**

**Figure 4F( p-ERK)**


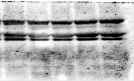


**p-ERK**

**44KD**

**42KD**

**34KD**

**55KD**

**72KD**

**Figure 6A (Raf)**


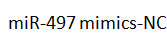


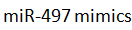

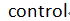


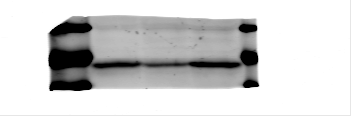


**72KD**

**55KD**

**95KD**

**Raf**

**75KD**

**Figure 6B (Total MEK)**

**
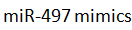

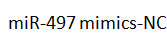

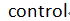
**


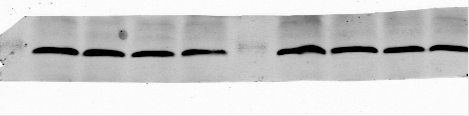


**34KD**

**26KD**

**Total-MEK**

**45KD**

**Figure 6C (p-MEK)**

**
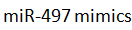

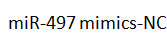

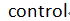
**

**
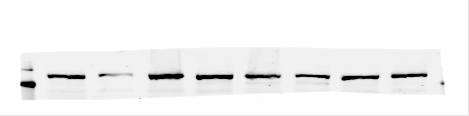
**

**p-MEK**

**45KD**

**34KD**

**55KD**

**26KD**

**Figure 6D (Total ERK )**

**
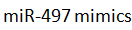

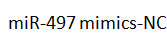
**

**
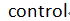
**

**Total-ERK**

**44KD**

**42KD**

**34KD**

**26KD**

**55KD**

**Figure 6E (p-ERK)**

**p-ERK**

**44KD**

**42KD**

**55KD**

**34KD**

**26KD**
